# Intravaginal Formulation Strategies for Vaginal Disorders: Optimizing Phytochemical and Biological Preparations—A Review

**DOI:** 10.3390/molecules31152676

**Published:** 2026-07-31

**Authors:** Ji Hye Hwang, Eun-Young Nam

**Affiliations:** 1 Department of Acupuncture & Moxibustion Medicine, College of Korean Medicine, Gachon University, Seongnam 13120, Republic of Korea; jhbori@nate.com; 2 Mimi Korean Medicine Clinic, Seoul 05667, Republic of Korea

**Keywords:** vaginitis, intravaginal preparations, vaginal drug delivery, herbal medicine, probiotics, bacterial vaginosis, vulvovaginal candidiasis, formulation optimization

## Abstract

Recurrent vaginal disorders remain a significant clinical challenge despite available antimicrobial and hormonal therapies. Therefore, natural, probiotic, and biological intravaginal preparations have been investigated as locally delivered adjunctive or supportive approaches for infectious, inflammatory, and atrophic vaginal conditions, with growing attention to how dosage form, mucosal retention, drug release, and patient acceptability influence clinical utility. This structured narrative review of 119 studies across six databases synthesizes clinical evidence for phytochemical-based and biological intravaginal preparations, organized into five formulation categories: creams and gels, suppositories and vaginal tablets, douches and washes, probiotic preparations, and combined phytochemical–biological preparations. Across formulation types, creams and gels were associated with application convenience and local symptom relief, whereas suppositories and vaginal tablets offered longer mucosal contact, dosing precision, and sustained exposure. Herbal intravaginal preparations were the most diverse, encompassing traditional medicine-derived creams, gels, suppositories, tablets, washes, and fumigation-based approaches. Among these, *Zataria multiflora*- and *Sophora flavescens*-based formulations were supported by comparatively consistent clinical signals. Probiotic preparations provided an important microbiome-directed model, whereas combined phytochemical–biological preparations remained preliminary. These findings support moving beyond active ingredient selection toward intravaginal formulation optimization. However, heterogeneity in formulations, comparators, outcome measures, and follow-up durations limited direct comparisons across studies.

## 1. Introduction

Vaginal dysbiosis and related mucosal conditions encompass a broad spectrum of recurrent or chronic gynecological problems characterized by altered vaginal microbiota, discharge, irritation, discomfort, and impaired quality of life. Representative conditions include bacterial vaginosis (BV), vulvovaginal candidiasis (VVC), atrophic vaginitis, and genitourinary syndrome of menopause (GSM), which differ in etiology but share overlapping clinical concerns related to local symptoms, recurrence, and mucosal vulnerability. BV accounts for approximately 29–49% of vaginitis cases among reproductive-aged women in the United States [1], whereas VVC affects up to 75% of women at least once during their lifetime, with recurrent VVC affecting an estimated 138 million women annually worldwide [2].

Current first-line treatments for infectious vaginitis predominantly rely on antibiotics and antifungals, including metronidazole, clindamycin, and fluconazole. Although these agents provide short-term symptom relief, they are associated with high recurrence rates. Approximately 50–80% of women with BV experience recurrence within 12 months of completing antibiotic therapy, with one landmark study reporting recurrence in 58–69% of women within 12 months of oral metronidazole treatment [3]. Repeated antibiotic use may further disrupt the vaginal microbiota by inhibiting the recolonization of beneficial *Lactobacillus* species, creating a cycle of recurrent infection. Hormonal therapies remain effective for postmenopausal women with atrophic vaginitis or GSM; however, they raise safety concerns in certain populations, including breast cancer survivors [4]. These limitations have prompted growing interest in alternative and complementary approaches to vaginal health management.

Natural and biological intravaginal preparations, including herbal extracts, essential oil-based formulations, phytoestrogen gels, and probiotic *Lactobacillus* products, have been increasingly investigated as locally delivered adjunctive or supportive approaches to vaginal health. These agents offer several theoretical advantages, including multi-target antimicrobial and anti-inflammatory mechanisms and, in the case of probiotic preparations, the potential to support the restoration of the vaginal microbiota [4,5,6,7]. Several randomized controlled trials (RCTs) have evaluated individual preparations across various vaginal conditions; however, the clinical evidence remains fragmented, with studies typically focusing on a single disease entity, ingredient, or formulation type in isolation [4,5,6,7,8,9]. Plant-derived phytochemicals constitute the principal bioactive components of many herbal preparations reviewed in this article. Their successful clinical translation depends not only on their intrinsic biological activity but also on formulation characteristics that influence local retention, controlled release, and bioavailability following intravaginal administration.

Existing reviews have largely examined herbal, probiotic, or phytoestrogenic interventions for individual conditions such as BV, VVC, or GSM, whereas few have integrated evidence across phytochemical-based and biologically active intravaginal formulations and delivery systems from a formulation-oriented perspective [1,5,6,8]. In addition, combined phytochemical–biological preparations have been reported in only a limited number of studies and remain insufficiently synthesized from a formulation-oriented clinical perspective [7,9,10]. This narrative review aims to provide a comprehensive overview of the available clinical evidence for natural and biological intravaginal preparations, organized by formulation type, and to identify frequently used ingredients, microbiome-related outcomes, patient acceptability considerations, and future research priorities from a formulation-optimization perspective. Unlike previous reviews that primarily summarize individual diseases, herbal ingredients, or probiotic interventions separately, the present review instead integrates evidence across intravaginal dosage forms to examine how formulation characteristics—including mucosal retention, release characteristics, local bioavailability, pH compatibility, and patient acceptability—influence the successful translation of plant-derived phytochemical formulations into intravaginal therapy.

## 2. Study Selection

This review was designed as a structured narrative review, combining comprehensive database searches with narrative synthesis to provide a formulation-oriented overview of the available clinical evidence. A literature search was performed across six databases: PubMed, EMBASE, Web of Science, Cochrane Library, Oriental Medicine Advanced Searching Integrated System (OASIS), Korea Institute of Oriental Medicine, and the China National Knowledge Infrastructure (CNKI). Searches were conducted without date restrictions for international databases, while CNKI searches were limited to publications from 2000 onward to focus on contemporary clinical evidence with more consistent indexing and full-text availability.

Searches combined terms across four thematic areas: vaginal conditions, natural or traditional medicine interventions, probiotic or biological preparations, and intravaginal dosage forms. Disease-related terms included vaginitis, BV, vulvovaginal candidiasis, vaginal atrophy, and GSM. Intervention-related terms included herbal medicine, plant extract, phytotherapy, natural product, traditional medicine, Chinese medicine, botanical, essential oil, phytoestrogen, probiotic, *Lactobacillus*, and synbiotic. Formulation-related terms included vaginal, intravaginal, suppository, gel, cream, pessary, wash, douche, tablet, and capsule. Equivalent controlled vocabulary and free-text terms were used where applicable in EMBASE and other databases. OASIS and CNKI were searched using Korean and Chinese terms for vaginitis, herbal external preparations, intravaginal administration, probiotic therapy, and fumigation therapy.

A total of 1793 records were identified across all databases (PubMed: 333; EMBASE: 696; Web of Science: 80; Cochrane Library: 18; OASIS: 68; and CNKI: 598). After the removal of 70 duplicates, 1723 records were screened based on their title and abstract. Studies were considered relevant if they addressed intravaginal or topical administration of natural or biological agents and clinical outcomes in women with vaginitis or related vaginal mucosal conditions. Eligible publication types included RCTs, non-randomized clinical trials, prospective observational studies, systematic reviews, and narrative reviews. Studies focusing exclusively on oral administration, in vitro or animal experiments, diagnostic methods, or vaginal microbiome profiling without therapeutic intervention were excluded. No formal language restrictions were applied; however, studies were primarily identified and assessed in English, Korean, and Chinese, reflecting the databases searched. Studies in other languages were retained when the full text was accessible and the content could be adequately assessed.

Following the full-text assessment, 119 studies were retained for narrative synthesis. The retained studies comprised RCTs and other clinical studies (*n* = 72) and systematic reviews, meta-analyses, and narrative reviews (*n* = 47). Studies were conducted across multiple geographic regions, with substantial contributions from Iran and the Middle East, East Asia (including China and Korea), Europe, and other regions. Given the heterogeneity of the study designs, interventions, and outcome measures, a narrative synthesis approach was adopted. Because this review was formulation-oriented rather than disease-oriented, the evidence was organized primarily by preparation type, rather than diagnosis, into creams and gels, suppositories and vaginal tablets, douches/washes and fumigation-based approaches, probiotic vaginal preparations, and combined phytochemical–biological preparations.

## 3. Formulation Types and Clinical Applications

The physicochemical properties of intravaginal dosage forms directly influence drug release kinetics, mucosal retention time, and patient acceptability and compliance. Table 1 summarizes the key characteristics of the major dosage forms represented within these categories, including physicochemical features, vaginal retention profiles, patient acceptability, potential limitations, and representative literature references.

### 3.1. Creams and Gels

Creams and gels were the most commonly investigated formulation types among natural vaginal preparations, identified in approximately 35 studies. The key clinical trials in this category are summarized in Table 2. These preparations offer practical advantages, including ease of application, broad mucosal contact, and high patient acceptability. VVC and BV were the most frequently targeted conditions in this category, with fewer studies addressing atrophic vaginitis and GSM.

#### 3.1.1. Herbal Creams and Gels for VVC and BV

*Zataria multiflora* Boiss., commonly known as Shirazi thyme, is an aromatic herb that is native to Iran, Afghanistan, and Pakistan. It was the most frequently studied single herbal ingredient in vaginal cream formulations for vaginitis. Three independent RCTs evaluated *Z. multiflora* vaginal preparations for different vaginitis subtypes. Simbar et al. [11] reported comparable clinical outcomes to metronidazole vaginal gel for BV in an RCT of 90 women (*n* = 45 per group), with no statistically significant difference in clinical cure rates based on the Amsel criteria between the groups. Khosravi et al. [12] reported clinical improvement with *Z. multiflora* cream for acute VVC, with white cheesy vaginal discharge positivity declining from 100% at baseline to 13.8% following treatment, alongside marked reduction in vulvar pruritus. Farshbaf-Khalili et al. [30] compared garlic, *Z. multiflora*, and clotrimazole vaginal cream for VVC prevention, with fungal culture positivity rates of 2.6%, 0%, and 0%, respectively, at 30 days post-treatment, suggesting comparable antifungal effects to the standard comparator.

*Prangos ferulacea* vaginal cream was evaluated in three RCTs across BV, VVC, and *Trichomonas vaginalis* infection [31,32,33]. Azadpour et al. [31] reported accelerated BV recovery when *P. ferulacea* cream was used as an adjunct to oral metronidazole, Motlagh et al. [32] reported outcomes comparable to metronidazole in *T. vaginalis* infection, and Ghiasi et al. [33] reported outcomes comparable to clotrimazole for VVC. Propolis-based vaginal creams were evaluated in two RCTs for both VVC and BV, with outcomes broadly comparable to standard antifungal and antibacterial comparators [34,35]. *Salvia officinalis* cream was compared with clotrimazole for VVC [36], and a subsequent RCT evaluated its potential role in preventing recurrent VVC with continued application [37]. Artemisia vaginal cream was compared with clotrimazole in a triple-blind RCT on VVC [38]. One RCT showed that curcumin-based vaginal cream showed outcomes comparable to clotrimazole for VVC [39]. A curcumin vaginal preparation showed greater improvement rates than metronidazole for BV (82% vs. 42%, *p* < 0.001) in an RCT [24]. Dill (*Anethum graveolens*) vaginal cream was evaluated against clotrimazole for VVC with comparable outcomes [40]. Henna (*Lawsonia inermis*) vaginal cream was compared with clotrimazole in an RCT for vaginal candidiasis [41]. A novel Mycozin herbal cream was compared with clotrimazole 1% in a triple-blind RCT [42]. Honey-based vaginal gel and yogurt-honey cream mixtures were associated with antimicrobial and symptomatic benefits for VVC [43,44].

Several cream or gel formulations were also evaluated for BV and related dysbiotic conditions. *Calendula officinalis* cream was evaluated against metronidazole for BV, with Pazhohideh et al. [45] reporting comparable overall outcomes in Amsel criteria improvement. *Quercus brantii* vaginal cream was evaluated against placebo for BV in an RCT by Zare et al. [23]. Avijgan et al. [27] evaluated a hydroalcoholic extract of *Echinophora platyloba* in comparison with fluconazole for chronic recurrent VVC and reported lower treatment failure rates (20% vs. 43.3%) and long-term recurrence rates (26.7% vs. 56.7%) than fluconazole alone. Romero-Cerecero et al. [46] conducted a randomized, double-blind, controlled pilot study of *Ageratina pichinchensis* extract for VVC, reporting a therapeutic success rate of 81.2% versus 86.6% for clotrimazole (*p* > 0.05), with a clinical efficacy of 94.1% and tolerability of 93.8% compared with 80.0% for the comparator. Khazaeian et al. [28] compared sucrose gel with metronidazole gel for BV symptom relief, with symptom improvement rates of 85.7% and 88.5%, respectively (*p* = 0.389), suggesting that sucrose gel may be a potential non-antibiotic option for symptom relief in BV. A *Cymbopogon olivieri*-based herbal vaginal product was evaluated for BV in a clinical trial [47]. Multi-Gyn ActiGel, which contains an aloe vera-derived 2QR-complex, was also evaluated for BV management [48].

Plant extract-based medical devices have also been investigated for the treatment of postmenopausal vulvovaginal symptoms. Galli et al. [29] evaluated a standardized class II medical device based on highly purified plant extracts, including *Zanthoxylum*, *Centella asiatica*, and tamarind, combined with hyaluronic acid over 150 days in women with postmenopausal vulvar and vaginal atrophy. The study reported significant improvements in the Vaginal Health Index and Female Sexual Distress Scale scores, with reductions in vaginal dryness and dyspareunia, supporting its potential as a non-hormonal option for postmenopausal vulvovaginal symptoms.

#### 3.1.2. Phytoestrogen Gels and Creams for Atrophic Vaginitis and GSM

For GSM and atrophic vaginitis, phytoestrogen-based creams and gels represented a clinically important subgroup because they may provide local symptom relief while avoiding systemic hormone exposure in specific populations. *Pueraria mirifica* gel was evaluated in three RCTs, with one placebo-controlled study showing significant improvement in objective vaginal microenvironmental parameters, including Nugent score and Vaginal Health Index, although subjective genitourinary symptoms did not significantly improve [13]. Fennel-containing vaginal creams, either alone or in combination with *S. officinalis* and flaxseed, were also associated with symptomatic improvement in postmenopausal vaginal atrophy, with a favorable local safety profile and no reported systemic adverse events [49,50]. Other plant-derived or phytoestrogenic vaginal preparations, including soy isoflavone gel, genistein, isoflavandiol E55-RS, nettle cream, and a Persian traditional medicine-based herbal gel, have been investigated in smaller clinical or proof-of-concept studies [51,52,53,54,55]. Systematic and narrative reviews have further summarized the broader phytotherapeutic, phytoestrogenic, and traditional medicine evidence base for vaginal atrophy and GSM [56,57,58,59]. Collectively, these findings suggest that plant-derived gels and creams may be useful non-hormonal or hormone-sparing options for treating GSM and atrophic vaginitis. However, future studies should prioritize validated patient-reported symptom outcomes, response durability, and formulation-specific tolerability.

### 3.2. Suppositories and Vaginal Tablets

Traditional herbal suppositories, pessaries, and vaginal tablets were among the earliest solid intravaginal dosage forms to be evaluated. Early Indian studies assessed a polyherbal pessary and Praneem polyherbal vaginal tablets for symptomatic vaginal discharge, providing early examples of traditional multi-ingredient solid intravaginal preparations [60,61]. Persian and Iranian traditional medicine-derived suppositories have also been evaluated in randomized trials, including the Forzejeh suppository for BV, myrtle–oak gall suppository for mixed and trichomonas vaginitis, and *Rheum turkestanicum* suppository for BV recurrence prevention [16,25,62]. Additional *Quercus*-, oak gall–ajwain-, and pomegranate-based vaginal tablets or suppositories showed symptomatic or microbiological improvements in vaginitis, VVC, or BV, with some studies reporting outcomes comparable to standard antifungal or antibacterial comparators [15,63,64].

Traditional Chinese medicine (TCM)-derived solid vaginal preparations included the Fufang FuRong effervescent suppository and a *Sophora flavescens*-based intravaginal film. Two studies of Fufang FuRong reported clinical improvement and restoration of vaginal microecological balance; notably, a randomized multicenter non-inferiority trial in 559 patients with mixed vaginitis showed non-inferiority to clindamycin phosphate cream, with comparable adverse event rates [26,65]. *S. flavescens* intravaginal film was also associated with favorable effectiveness and pharmacoeconomic outcomes in postpartum vaginal dysbiosis [66]. Other traditional solid formulations, including plant extract-based vaginal suppositories, a *Viola odorata* suppository, Dhatakyadi Varti, and an oregano–lemon balm herbal vaginal tablet, further support the clinical exploration of solid herbal vaginal dosage forms across different traditional medicine systems [17,67,68,69]. Selected clinical trials in this category are included in Table 2.

### 3.3. Douches and Washes

Vaginal douche and wash preparations were identified in eight studies. Selected clinical trials in this category are summarized in Table 2. This category encompasses both Western-style herbal washes and the traditional East Asian practices of fumigation and medicated sitz baths. Although non-therapeutic vaginal douching has been associated with disruption of the vaginal microbiota in observational studies, the herbal wash preparations reviewed here were specifically formulated with therapeutic intent and evaluated in clinical or traditional medicine contexts. Future studies should clearly distinguish between standardized therapeutic intravaginal wash formulations and routine or cosmetic douching practices.

#### 3.3.1. Herbal Vaginal Washes and Douches

Leite et al. [19] conducted an RCT comparing a Brazilian pepper tree (*Schinus*) extract with metronidazole gel for BV, reporting comparable microbiological and clinical outcomes. Zeinab et al. [20] compared *Q. brantii* vaginal douching with clotrimazole for VVC and found comparable antifungal outcomes. Guinot et al. [70] evaluated a non-soap herbal-based intimate hygiene solution (Zelesse) for acceptability, tolerability, and effects on the signs and symptoms of vulvovaginitis, reporting favorable tolerability and symptom-related outcomes.

#### 3.3.2. Fumigation and Medicated Sitz Bath (East Asian Tradition)

Fumigation therapy and medicated sitz baths are traditional East Asian therapeutic modalities in which herbal decoctions are administered as steam or warm immersions to the perineal and vulvovaginal areas. Unlike conventional intravaginal dosage forms, these approaches provide transient external or vestibular mucosal exposure and may be associated with heat-mediated local circulation effect and symptom relief. However, their mechanistic basis and clinical relevance require further validation.

Two Korean systematic reviews examined this evidence base. Baek et al. [21] conducted a systematic review of fumigation therapy for atrophic vaginitis, identifying five RCTs that reported improvement trends in treatment response, although three studies did not reach statistical significance. Park et al. [22] reviewed herbal medicine fumigation treatment for mycotic vaginitis across five RCTs, reporting favorable effects compared with control treatments, although methodological limitations should be considered. Hwang et al. [71] analyzed RCTs of Korean medicine (KM) external treatments for BV. Wu et al. [72] evaluated Wandai Decoction combined with TCM fumigation and washing in patients with chronic vaginitis following sintilimab treatment for small cell lung cancer, representing an application of combined internal and external herbal therapy in an immunocompromised oncological context. Korean in vitro studies have also identified several heat-clearing or aromatic dampness-resolving herbal ingredients with antibacterial activity against vaginal pathogens, including *Gardnerella vaginalis* [73,74,75]. These findings may provide a mechanistic context for traditional external herbal preparations; however, clinical validation is necessary.

### 3.4. Probiotic Vaginal Preparations

Probiotic vaginal preparations, primarily containing *Lactobacillus* species administered directly into the vaginal canal, were identified in 21 clinical studies, in addition to numerous systematic reviews and meta-analyses. The key clinical trials in this category are summarized in Table 3. Nader-Macías et al. comprehensively reviewed advances in the knowledge and clinical applications of lactic acid bacteria as probiotics in the urogenital tract, laying the foundation for this biotherapeutic approach [76]. *Lactobacillus crispatus*, *L. rhamnosus*, *L. plantarum*, and *L. fermentum* were the most frequently investigated strains.

#### 3.4.1. *Lactobacillus* Vaginal Capsules and Tablets for BV

*Lactobacillus* vaginal capsules, tablets, and suppositories have been evaluated for BV treatment and recurrence prevention. Early studies established proof of concept for vaginal lactobacilli supplementation, although the outcomes were heterogeneous. For example, Eriksson et al. [88,89] found no significant improvement in cure rates after clindamycin treatment followed by vaginal lactobacilli, suggesting that strain selection, treatment timing, and dosage form may influence clinical outcomes. Subsequent RCTs reported more favorable findings. Ya et al. [81,90] reported lower BV recurrence with vaginal probiotic capsules than placebo at 2 months (15.8% vs. 45.0%; odds ratio 0.23, *p* < 0.001), and Larsson et al. [78] reported improved 6-month BV-free rates after clindamycin followed by human lactobacilli compared with placebo (64.9% vs. 46.2%, *p* = 0.027). Additional studies evaluated vaginal *Lactobacillus* preparations after metronidazole treatment in postpartum and pregnancy-associated vaginitis, although the formulations and target populations varied [91,92,93,94,95].

Comparatively strong clinical evidence comes from Lactin-V (*L. crispatus* CTV-05), which reduced BV recurrence after metronidazole in a phase 2b trial over 24 weeks (relative risk (RR) 0.66, 95% confidence interval (CI) 0.44–0.87; *n* = 228), with subsequent studies reporting microbiological and immunological findings in higher-risk populations [77,96,97,98]. Further trials extended the evidence to asymptomatic BV, intermediate vaginal flora, women with human immunodeficiency virus, and surgically menopausal women [79,80,99,100]. However, not all studies showed significant recurrence prevention, indicating that probiotic effects are likely influenced by strain specificity, host context, timing after antimicrobial therapy, and formulation characteristics such as mucosal retention and sustained exposure.

#### 3.4.2. *Lactobacillus* Vaginal Preparations for VVC

Vaginal probiotic preparations have mainly been investigated as adjunctive or recurrence-preventive therapies for VVC. Slow-release vaginal probiotic products, soft capsules, gels, and adjunctive capsules with antifungal therapy have shown potential benefits in symptom control, microbiological eradication, recurrence reduction, and tolerability, although the evidence remains more heterogeneous than for BV [101,102,103,104,105,106]. Among the more clinically relevant findings, *Limosilactobacillus fermentum* LF5 showed microbiological eradication comparable to topical miconazole, with fewer local adverse reactions, and vaginal probiotic administration appeared more favorable than oral administration as an adjunct to fluconazole in a comparative study [83,107]. Reviews have further discussed vaginal microbiota restoration and the potential role of both *Lactobacillus* and non-*Lactobacillus* probiotic approaches in women’s health [6,7,108,109].

#### 3.4.3. Gynoflor: A Model Combination Biological–Hormonal Preparation

Gynoflor, a combination of *L. acidophilus* and low-dose estriol, is a well-studied biological–hormonal vaginal preparation. In BV, Gynoflor showed short-term equivalence to metronidazole in a pilot RCT, although lower lactobacillary outcomes at follow-up suggested a possible maintenance rather than primary-treatment role [85]. In postmenopausal vaginal atrophy, clinical studies reported symptomatic improvement, including investigator-rated and patient-reported improvement after treatment [110]. A pharmacokinetic safety study in postmenopausal patients with breast cancer receiving aromatase inhibitors found no increase in systemic estradiol or estrone levels while improving the vaginal maturation index, supporting its potential local safety in estrogen-sensitive populations [86]. Additional studies reported improvements in sexual quality of life, and a clinical review further summarized its therapeutic profile [111,112].

#### 3.4.4. Other Probiotic and Live Biotherapeutic Preparations

Emerging biotherapeutic preparations further expand the formulation landscape, including autochthonous lactobacilli capsules, *Lactobacillus* suppositories, *L. crispatus*-containing products, *L. rhamnosus*-based adjuvant therapy, and oral-versus-vaginal probiotic comparisons [82,113,114,115,116,117,118,119]. Several studies reported improvements in Nugent score, Vaginal Health Index, microbiota restoration, adherence, and tolerability, although the findings varied by strain, route, and population. A large three-arm RCT found no additional recurrence-prevention benefit when an estrogen-containing vaginal probiotic or vaginal clindamycin was added to oral metronidazole, underscoring that probiotic efficacy depends on strain, timing, host context, and formulation characteristics rather than probiotic use alone [84].

### 3.5. Combined Phytochemical–Biological Vaginal Preparations

**Figure 1:**
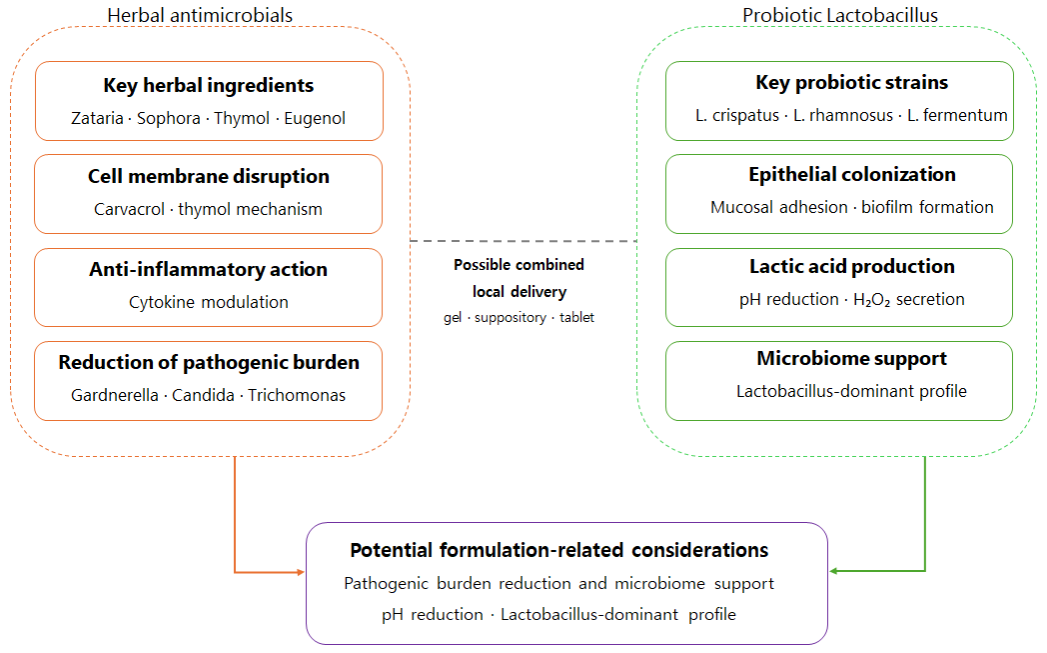
Conceptual overview of formulation considerations for combined phytochemical–biological vaginal preparations. This figure is hypothesis-generating and does not imply the established clinical superiority of combined or synbiotic formulations. Phytochemical components may provide local antimicrobial or anti-inflammatory activity, whereas probiotic components may support *Lactobacillus*-dominant vaginal microbiota. Potential formulation considerations include component compatibility, mucosal retention, release profile, local tolerability, and patient acceptability. Dedicated clinical studies are required to determine whether such combined approaches provide advantages over single-component formulations.

Combined phytochemical–biological vaginal preparations were identified in a small number of clinical studies. These preparations combine antimicrobial or prebiotic components with probiotic or microbiome-modulating agents; however, the available evidence remains preliminary and should not be interpreted as establishing the clinical superiority of combination therapy. Selected clinical studies in this category are included in Table 3. Patel et al. compared a polyherbal pessary with Ginlac-V, a conventional combination containing clotrimazole, tinidazole, and *Lactobacillus*, for symptomatic vaginal discharge, reporting comparable clinical outcomes and providing an early example of combined intravaginal delivery [60]. Tena González et al. evaluated boric acid with probiotics for bacterial and fungal vulvovaginitis, suggesting that antimicrobial and microbiome-supportive components may be incorporated within a local vaginal treatment strategy [120]. Murina et al. evaluated a medical device containing thymol, eugenol, *Lactobacillus fermentum* LF10, *L. plantarum* LP02, and galactooligosaccharides for BV and VVC; microbiological normalization was reported in the BV, VVC, and recurrent VVC subgroups [87]. Recently, Ravel et al. evaluated a mucoadhesive slow-release *L. crispatus*-based vaginal synbiotic tablet and reported a more frequent conversion to a *Lactobacillus*-dominant microbiome than with placebo and other delivery formats [18]. Overall, these studies provide early formulation-level insights into component compatibility, mucosal retention, and controlled release in combined phytochemical–biological vaginal preparations. Related review literature has also discussed intravaginal prebiotic delivery, interactions between *Lactobacillus* and herbal components, probiotic gel delivery platforms, and personalized approaches to vaginal co-infections [121,122,123,124]. A conceptual overview of the formulation considerations for these combined preparations is presented in Figure 1.

## 4. Frequently Identified Herbal Ingredients

Across the included studies, several herbal ingredients and probiotic strains appeared repeatedly across multiple formulation types, geographic regions, and research groups, suggesting recurring clinical and formulation interest in specific natural and biological vaginal preparations. The key phytochemicals identified in the included studies, their botanical sources, putative mechanisms of action, and target conditions are summarized in Table 4.

### 4.1. Zataria multiflora

*Z. multiflora* Boiss. was the most frequently studied single herbal ingredient in vaginal cream formulations. Three independent RCTs evaluated *Z. multiflora* in VVC and BV, reporting outcomes broadly comparable to metronidazole for BV and clotrimazole for VVC in selected clinical endpoints [11,12,30]. A systematic review by Najafi et al. further summarized clinical studies on herbal medicines for BV, including *Z. multiflora*, and reported outcomes comparable to metronidazole in the included trials [5]. The primary active constituents, including carvacrol, thymol, and p-cymene, have been associated with broad-spectrum antibacterial and antifungal activities through membrane-disruptive mechanisms. These findings support *Z. multiflora* as a frequently investigated herbal ingredient for further formulation-oriented clinical research.

### 4.2. Sophora flavescens

*S. flavescens* Aiton (Kushen), rich in matrine and oxymatrine, was the most comprehensively evaluated TCM-derived ingredient for vaginal preparations. Meta-analytic evidence showed that Kushen gel produced greater symptom improvement than metronidazole with fewer adverse events [14], while additional studies supported its use as an adjunctive antifungal treatment for VVC and as an intravaginal film for postpartum vaginal dysbiosis [66,125]. In a network meta-analysis of nine topical Chinese polyherbal preparations for VVC, *S. flavescens*-based preparations were among the most frequently evaluated and ranked fourth in terms of negative conversion rates [126]. Collectively, these findings suggest that *S. flavescens* is a well-supported herbal ingredient for further formulation-oriented clinical research. Beyond *S. flavescens*, several East Asian and KM reviews provide broader contextual evidence for herbal intravaginal or external treatments in vaginal disorders. These include systematic reviews of traditional East Asian herbal medicine for atrophic vaginitis, intravaginal oriental medicine for menopausal vaginal atrophy, KM treatment trends for vaginitis during pregnancy, external KM treatments for BV, and fumigation therapy for atrophic or mycotic vaginitis [21,22,71,127,128,129]. In parallel, a recent expert council resolution on probiotics for vaginal and intestinal dysbiosis reflects the growing professional consensus regarding biological vaginal preparations in gynecological practice [9].

### 4.3. Pueraria mirifica

*P. mirifica* Airy Shaw & Suvatabhandu, a phytoestrogen-rich herb, was the most studied herbal ingredient for managing GSM and atrophic vaginitis. Three clinical studies evaluated *P. mirifica* vaginal gel, demonstrating significant improvements in objective vaginal microenvironmental parameters, including Nugent score normalization (6.7% vs. 23.3%, *p* = 0.006) and Vaginal Health Index (increase: 2.12 vs. 0.81, *p* = 0.016) [13]. However, the effect on patient-reported genitourinary symptoms was not demonstrated, suggesting that objective microenvironmental changes may not necessarily translate into patient-reported symptom improvement. Therefore, future trials should incorporate validated symptom-focused endpoints and longer follow-up periods. The phytoestrogenic activity attributed to miroestrol and deoxymiroestrol makes *P. mirifica* preparations particularly relevant for populations in which systemic hormonal therapy carries an elevated risk.

### 4.4. Foeniculum vulgare (Fennel)

*F. vulgare* Mill. (fennel) was evaluated for atrophic vaginitis management in combination herbal formulations, with trans-anethole considered a putative phytoestrogenic component. These studies reported symptomatic improvement and favorable local tolerability, although long-term safety and systemic hormonal effects require further evaluation [49,50].

### 4.5. Quercus-Derived Preparations

Oak-derived preparations appeared across multiple formulation types and vaginitis subtypes: vaginal cream for BV [23], vaginal douche versus clotrimazole for VVC [20], herbal vaginal tablet with ajwain [63], and fruit hull suppositories for VVC [15]. The antimicrobial activity attributed to tannins, gallic acid, and ellagic acid supports the consistent interest in *Quercus* as a versatile herbal antimicrobial candidate.

### 4.6. Essential Oil Constituents and Lactobacillus Strains

Thymol, carvacrol, and eugenol appeared repeatedly in preparations from diverse geographic and botanical origins. These essential oil constituents share antimicrobial mechanisms related to membrane disruption and may be relevant for formulation compatibility when used alongside probiotics or microbiome-modulating components. Among probiotic strains, *L. crispatus* CTV-05 (Lactin-V) showed the strongest clinical evidence for BV recurrence prevention, including a phase 2b trial reporting reduced recurrence risk [77]. *L. rhamnosus* was evaluated in multiple BV recurrence-prevention studies [78,80,91], whereas *L. fermentum* and *L. plantarum* strains were evaluated in selected VVC or recurrence-prevention studies [102,105].

Cochrane reviews on probiotics for BV and VVC concluded that the evidence was insufficient to recommend probiotics as standalone primary therapy [1,8], highlighting the need for larger, well-designed trials and formulation-specific evaluation. Selected systematic reviews and meta-analyses further contextualized the clinical evidence for herbal, probiotic, and combined approaches, including medicinal plants for BV [130], Kushen gel, topical Chinese polyherbal preparations, fumigation therapy, and antibiotics combined with intravaginal probiotics [131] (Table 5). Broader reviews of vaginal microbiota and probiotic interventions have also emphasized the importance of strain specificity, host context, and targeted microbiome-supportive strategies [132,133].

## 5. Discussion

Plant-derived phytochemicals constituted the principal active agents identified across most formulation categories included in this review, supporting the central focus of the present synthesis. This narrative review identified a geographically diverse body of clinical evidence for natural and biological vaginal preparations across vaginitis subtypes and formulation categories. A total of 119 studies were retained, comprising 72 clinical studies and 47 systematic reviews, meta-analyses, and narrative reviews, suggesting that natural and biological intravaginal preparations have been increasingly investigated as potential local therapeutic options.

### 5.1. Formulation Type as a Clinically Relevant Variable

The formulation type emerged as a clinically relevant variable that may influence clinical performance, independent of the active ingredient. Creams and gels were the most extensively studied dosage forms, with several preparations reporting outcomes broadly comparable to standard treatments. However, their relatively short mucosal contact time may limit their therapeutic duration compared with suppository- or tablet-based delivery. Suppositories and vaginal tablets may partly address this limitation through extended retention and controlled drug release, whereas slow-release probiotic tablets represent a potential approach for sustained mucosal exposure. Vaginal wash and fumigation preparations occupy a distinct clinical niche. East Asian fumigation therapy may provide heat-mediated local exposure, mucosal contact, and local circulation-related effects that could be relevant for atrophic vaginitis, although these mechanisms require further investigation. Although non-therapeutic vaginal douching has been associated with microbiome disruption, standardized therapeutic herbal wash preparations evaluated in controlled or traditional medicine contexts should be distinguished from routine hygiene-related douching. The diversity of intravaginal drug delivery systems and their respective pharmacokinetic profiles have been reviewed, providing a framework for considering formulation selection according to therapeutic goals and target conditions [134].

Beyond formulation type alone, several pharmaceutical principles may further influence intravaginal drug performance. Vaginal pH, which differs between reproductive-age and postmenopausal women, may affect drug ionization, solubility, diffusion, and release characteristics, while mucoadhesive excipients (e.g., carbopol and hydroxypropyl methylcellulose) can prolong mucosal residence time, influence local drug absorption and diffusion, and enhance local drug exposure. Controlled-release systems and appropriate excipient compatibility are particularly relevant for phytochemical and probiotic formulations, where sustained drug delivery, preservation of probiotic viability and biological activity, and formulation stability should all be considered during product development.

Direct comparison across formulation types was limited by substantial heterogeneity in follow-up duration, which ranged from 2 to 24 weeks across the included studies, as well as differences in outcome definitions, diagnostic criteria, and comparator treatments. These variations precluded meaningful pooled comparison of cure and recurrence rates and should be considered when interpreting apparent similarities in clinical performance across formulations.

### 5.2. Evidence Convergence Regarding Key Herbal Ingredients

Several herbal ingredients appeared repeatedly across multiple research groups, geographic regions, and formulation types. *Z. multiflora* was evaluated in three independent RCTs across VVC and BV [11,12,30], and a systematic review of 13 studies reported outcomes generally comparable to metronidazole for BV [5]. *S. flavescens* emerged as one of the most frequently evaluated TCM-derived ingredients, with meta-analytic evidence suggesting greater symptom improvement than metronidazole (RR = 1.13) and fewer adverse events (RR = 0.29) [14]. In a network meta-analysis of topical Chinese polyherbal preparations for VVC, *S. flavescens*-based preparations were among the most frequently evaluated options and ranked fourth for negative conversion rates among nine preparations [126]. Together, these findings suggest that *S. flavescens* is one of the better-supported herbal ingredients for further formulation-oriented clinical research. In addition to these two extensively documented ingredients, several other phytochemicals showed recurring clinical interest. *Quercus*-derived preparations appeared across four formulation types and three vaginitis subtypes, with antimicrobial activity attributed to tannins, gallic acid, and ellagic acid [15,20,23,63]. *P. ferulacea* was evaluated for BV, trichomoniasis, and VVC in three RCTs [31,32,33], suggesting potential cross-indication relevance that requires confirmation. Curcumin preparations showed higher complete improvement rates than metronidazole for BV in one randomized trial (82% vs. 42%, *p* < 0.001) [24], a finding that warrants replication in larger trials. Collectively, these phytochemicals possess bioactive properties relevant to vaginal health, including antimicrobial and anti-inflammatory activities. Some may also have potential *Lactobacillus*-sparing properties, which could be relevant to compatibility-guided formulation design, although this remains to be confirmed in future clinical studies. Recent in vitro phytochemical research has also demonstrated inhibitory activity of phenolic-rich *Glycyrrhiza glabra* root extract against *Candida albicans* and several bacterial species, supporting further investigation of chemically well-characterized plant extracts for local antimicrobial formulation development [135].

### 5.3. Korean and East Asian External Herbal Therapy: A Distinct Evidence Tradition

Korean and broader East Asian external herbal therapy represent a distinct evidence stream that has been less visible in English-language vaginitis literature. Fumigation therapy and medicated sitz baths have been reviewed in the KM literature, with Baek et al. identifying five RCTs of fumigation therapy for atrophic vaginitis and Park et al. reviewing five RCTs of fumigation therapy for mycotic vaginitis [21,22]. These modalities may involve heat-mediated local exposure, mucosal contact, and local circulation-related effects, which could be relevant for symptom management in atrophic vaginitis; however, these proposed mechanisms require further validation. In vitro studies by Korean research groups have also reported the inhibitory effects of heat-clearing and aromatic dampness-resolving herbal formulations on vaginal pathogens, including *G. vaginalis* [73,74,75]. One study reported partial synergism between Gagam-seopyoungjeon and clindamycin, suggesting a possible basis for future studies on adjunctive strategies, although clinical confirmation is still needed [75]. The relative absence of this evidence base from the global vaginitis literature represents both a limitation and an opportunity. Integration of rigorously conducted East Asian external herbal therapy trials into international evidence synthesis may broaden the range of formulation approaches considered in future clinical research.

### 5.4. Combined Phytochemical–Biological Preparations: Formulation Considerations

Combined phytochemical–biological preparations remain an early-stage formulation category in the current clinical literature. These preparations may incorporate antimicrobial, prebiotic, probiotic, or microbiome-modulating components within a single local delivery system; however, direct clinical evidence remains limited to a small number of studies and should not be interpreted as establishing clinical superiority over single-component formulations. Murina et al. evaluated a medical device combining thymol, eugenol, two *Lactobacillus* strains, and a prebiotic, reporting microbiological normalization in BV, VVC, and recurrent VVC subgroups [87]. The VS-01 study further illustrates the relevance of prebiotic substrate formulation and slow-release mucoadhesive delivery for intravaginal biological preparations [18], although it should not be interpreted as direct evidence for traditional herbal–probiotic combination therapy. Gynoflor also illustrates the feasibility of integrating biological and pharmacological components within a single vaginal delivery system, while follow-up findings suggest that probiotic-containing preparations may require careful positioning according to the treatment phase and clinical context [110]. In vitro evidence indicating that plant-derived polyphenols may exert antimicrobial and prebiotic-related effects, and that selected *Lactobacillus* strains combined with plant extracts may show enhanced antimicrobial activity against *Candida*, provides mechanistic rationale for further formulation-oriented studies [136,137].

### 5.5. Probiotic Evidence: From Supplementation to Biotherapy

The evidence base for vaginal probiotic preparations has increasingly shifted toward more rigorous clinical trial designs. The phase 2b Lactin-V trial reported a reduced cumulative risk of BV recurrence with *L. crispatus* CTV-05 compared with placebo (RR 0.66, 95% CI 0.44–0.87) in 228 randomized women [77]. Earlier work by Ya et al. reported lower BV recurrence with vaginal probiotic capsules than with placebo [81]. Meta-analytic evidence further suggests that antibiotics combined with intravaginal probiotics may reduce short-term BV recurrence at 12–16 weeks, although longer-term benefits require further investigation [131,138]. Together, these findings suggest that intravaginal probiotic delivery may be clinically relevant for BV recurrence prevention, particularly when strain selection, timing after antimicrobial therapy, and formulation characteristics are carefully considered.

### 5.6. Geographic and Methodological Considerations

A notable geographic concentration was observed in the clinical evidence base. Iranian and Persian traditional medicine research contributed many of the herbal cream and gel RCTs, with *Z. multiflora, P. ferulacea*, and propolis preparations predominantly investigated in this region. TCM and KM research contributed to the evidence on traditional suppositories, washes, and external therapies, including the Fufang FuRong non-inferiority trial and KM studies on fumigation therapy. European and North American studies provided much of the evidence in the probiotic and biotherapeutic categories, including Lactin-V and Gynoflor studies. This geographic distribution reflects differing traditional medicine frameworks, regulatory environments, and research priorities, suggesting that cross-cultural validation remains limited. For example, *Z. multiflora* has been primarily studied in Iran, whereas *L. crispatus* CTV-05 formulations have been mainly evaluated in North American populations. Future international collaborative trials would improve the generalizability of the current evidence base.

### 5.7. Clinical Implications and Patient-Centered Considerations

The evidence reviewed here supports a patient-centered approach to intravaginal formulation selection. Formulation choice should be guided not only by the active ingredient or strain but also by the intended delivery site, mucosal residence time, dosing accuracy, local tissue condition, patient comfort, cultural acceptability, and access to standardized preparations. For recurrent BV, sustained or sequential intravaginal probiotic delivery has relatively strong clinical support among biological preparations, as illustrated by the Lactin-V trial and the Larsson et al. clindamycin–lactobacilli sequential regimen [77,78]. For acute VVC, selected herbal preparations such as *Z. multiflora* and *S. flavescens* have reported outcomes broadly comparable to standard antifungal comparators in clinical studies, although further confirmatory trials are required [11,12,14,30]. Combined phytochemical–biological preparations remain preliminary formulation concepts, and their clinical development will require careful attention to component compatibility, probiotic viability, and optimized mucosal delivery [18,87].

The formulation type itself may influence patient acceptability and adherence, independent of the active ingredient. Suppositories and vaginal tablets offer dosing accuracy and prolonged mucosal retention, making them suitable for deep vaginal delivery and overnight use; however, in patients with GSM, atrophic changes, vaginal dryness, or mucosal fragility, insertion may cause discomfort and reduce adherence. In such cases, cream or gel formulations may be better tolerated, and bioadhesive gels may provide additional benefits through moisturizing effects and prolonged mucosal contact. Aqueous wash and douche preparations offer ease of use and broad coverage but provide only transient mucosal contact, suggesting a more adjunctive role rather than primary treatment. Traditional East Asian fumigation preparations represent a non-insertion modality with cultural familiarity in specific populations, although their evidence base remains limited, and their effects are likely concentrated in the vulvar and vestibular areas. These formulation-specific considerations should inform individualized therapeutic decisions alongside efficacy data.

These findings highlight that the clinical translation of plant-derived phytochemicals depends not only on their biological activity but also on formulation characteristics that influence local delivery, mucosal retention, pH compatibility, patient acceptability, and clinical performance, as illustrated by the clinical evaluation of a *Pueraria mirifica* vaginal gel [13]. Patient-reported outcomes, including ease of application, insertion comfort, leakage, odor, and treatment acceptability, were inconsistently reported across the included studies, limiting direct comparison of patient experience across formulation types. Future clinical trials should incorporate validated patient-reported outcome measures to better evaluate patient experience, adherence, and real-world usability across formulation types.

## 6. Future Research Priorities

Several research priorities emerge from this review. First, phytochemical standardization of herbal intravaginal preparations is important for reproducibility and regulatory evaluation. Future trials should report active-marker concentrations, extraction methods, batch-to-batch variability, and stability data. Standardization should also consider how raw-material selection and processing conditions may influence contaminant control and final product safety. Although not specific to medicinal extracts or intravaginal products, processing studies in plant-derived materials illustrate the importance of validating processing-related quality-control procedures [139]. Second, comparative effectiveness trials across formulation types for the same indication are needed to clarify whether creams, gels, suppositories, tablets, washes, or slow-release delivery systems influence outcomes independent of the active ingredient. Third, longer follow-up periods, extending to at least 12 months and, where feasible, up to 24 months, are needed to assess recurrence prevention, which is one of the most clinically relevant endpoints for BV, VVC, and recurrent vaginal dysbiosis. Fourth, vaginal microbiome sequencing endpoints should be incorporated alongside traditional clinical cure criteria to clarify whether symptomatic improvement is accompanied by durable microbiome changes or the maintenance of *Lactobacillus*. Selected probiotic formulations have demonstrated clinical efficacy, including a reduced recurrence of bacterial vaginosis following intravaginal Lactin-V administration [77]. However, probiotics are retained in this review as microbiome-supportive interventions and clinically relevant comparators rather than as primary phytochemical agents.

Finally, combined phytochemical–biological vaginal preparations warrant further investigation as an emerging formulation category. Although recent clinical studies suggest that integrating antimicrobial, prebiotic, or probiotic components into local vaginal delivery systems may be feasible in selected patients with mixed or recurrent vaginitis [18,87], current evidence remains limited. Future randomized trials should evaluate standardized phytochemical extracts, defined *Lactobacillus* strains, probiotic viability, optimized dosing schedules, mucosal retention characteristics, tolerability, and microbiome-related outcomes within clearly specified intravaginal formulation platforms. Beyond currently available phytochemical formulations, plant-derived antimicrobial technologies may also provide additional directions for future formulation research. Green-synthesized silver nanoparticles produced using *Rhus coriaria* agricultural by-products have demonstrated in vitro activity against *C. albicans* and both Gram-positive and Gram-negative bacteria. Such approaches may warrant further investigation, although their suitability for intravaginal application, mucosal safety, and clinical use remains to be established [140].

## 7. Limitations

Despite searching six databases, including CNKI, East Asian clinical literature on herbal vaginal preparations may still be underrepresented. The heterogeneity of study designs, intervention compositions, dosing regimens, and outcome measures precluded quantitative synthesis. Moreover, the broad inclusion of multiple vaginitis subtypes and related vaginal conditions, while enabling a comprehensive formulation-oriented overview, introduced heterogeneity in diagnostic criteria, outcome measures, and comparator treatments across the retained studies. This limits direct cross-condition comparisons; BV and VVC were supported by the largest number of studies, whereas trichomoniasis, mixed vaginitis, and GSM had comparatively limited representation in the retained literature. As this was a structured narrative rather than a systematic review, potential selection bias cannot be excluded, and the methodological quality of the retained studies was not formally assessed. Additionally, several included RCTs had relatively small sample sizes, often enrolling fewer than 100 participants, and employed heterogeneous diagnostic criteria, including Amsel criteria, Nugent scoring, and culture-based methods, thereby limiting cross-study comparability. For probiotic preparations, potential sponsorship-related bias could not be fully evaluated because funding and manufacturer involvement were not consistently reported across studies. Short follow-up periods in many RCTs also limit conclusions regarding long-term efficacy and recurrence prevention. Furthermore, many clinical trials evaluating herbal preparations lacked rigorous phytochemical standardization and detailed reporting of active-marker concentrations, which challenges reproducibility. Variations in probiotic strain viability and product stability across different clinical settings were also not consistently reported, representing a broader challenge for the clinical translation of *Lactobacillus*-based biotherapeutics [141]. Finally, many herbal studies lacked dose–response characterization and formulation-specific pharmacological data, limiting direct translation into clinical dosing recommendations and underscoring the need for more rigorous phytopharmacological evaluations in future trials. Although a formal evidence-grading system was not applied, the available clinical evidence differed in strength across individual interventions. Among probiotic formulations, Lactin-V is supported by one large, well-designed randomized controlled trial demonstrating reduced BV recurrence; however, confirmation in independent populations and longer-term implementation studies remains limited. Clinical evidence for Gynoflor in postmenopausal women with GSM is based on a limited number of clinical studies, including a randomized trial and a pharmacokinetic safety study, with generally favorable findings regarding vaginal symptoms and local tolerability; however, the evidence base remains relatively small, and broader multicenter validation is needed. Among herbal preparations, *Zataria multiflora* has been evaluated in several randomized clinical studies with generally favorable findings compared with conventional antifungal therapy; however, most studies were conducted within limited geographic regions and involved relatively modest sample sizes, warranting further external validation.

## 8. Conclusions

Plant-derived phytochemical-based and biological intravaginal preparations represent a growing area of clinical investigation, spanning herbal formulations, phytoestrogen-based products, probiotic *Lactobacillus* preparations, and combined phytochemical–biological approaches. This structured narrative review suggests that these preparations may have clinical relevance across BV, VVC, atrophic vaginitis, GSM, and related vaginal conditions, particularly when formulation type, mucosal retention, local tolerability, and microbiome-related effects are considered in relation to clinical context. Formulation type appears to be a clinically relevant variable. Creams and gels represented the broadest clinical evidence base, suppositories and vaginal tablets may offer advantages in dosing accuracy and mucosal retention, and slow-release or mucoadhesive systems may support prolonged local exposure. East Asian fumigation and wash-based approaches represent distinct external or local delivery modalities, although their mechanisms and clinical roles require further validation. Among herbal ingredients, *Z. multiflora, S. flavescens, P. mirifica*, and *Quercus*-derived preparations were repeatedly identified across clinical studies or reviews, suggesting recurring interest in these candidates for further formulation-oriented research. Probiotic preparations, particularly *L. crispatus*-based products, showed clinically relevant evidence for BV recurrence prevention, while combined phytochemical–biological preparations remain preliminary and should be interpreted as formulation concepts rather than established, superior therapies. Future research should prioritize phytochemical standardization, strain-specific probiotic characterization, longer follow-up, validated patient-reported outcomes, microbiome-related endpoints, and direct comparison of formulation types within the same clinical indication. Overall, shifting attention from active ingredients alone toward formulation optimization, mucosal delivery characteristics, patient acceptability, and standardized preparation quality may help refine the clinical application of natural and biological intravaginal therapies for recurrent and chronic vaginal disorders. This review further emphasizes that the successful clinical translation of plant-derived phytochemicals depends not only on their bioactive constituents but also on formulation strategies that optimize local delivery, mucosal retention, and patient acceptability within the vaginal environment.

## Figures and Tables

**Table 1:** Key physicochemical characteristics and clinical utility of intravaginal dosage forms.

Dosage Form	Key Physicochemical Characteristics	Vaginal Retention	Patient Acceptability and Compliance	Potential Limitations	Representative References
Cream	O/W emulsion; broad spreadability; pH-sensitive formulations applicable	Moderate (leakage possible)	Easy application; lower insertion burden than solid dosage forms	Shorter residence time; potential leakage affecting compliance	Simbar 2008 [11]; Khosravi 2008 [12]
Gel	Bioadhesive polymer-based (e.g., carbopol, HPMC); sustained mucosal contact	High (prolonged by viscosity)	Comfortable; suitable for GSM/atrophic vaginitis	Requires appropriate viscosity; stability concerns for some formulations	Sritonchai 2020 [13]; Lan 2015 [14]
Suppository	Melting base (e.g., cocoa butter, PEG); body temperature dissolution	High (local release upon melting)	Accurate dosing; overnight use preferred	Insertion discomfort in mucosal fragility; rapid melt may reduce retention	Simbar 2026 [15]; Askari 2020 [16]
Vaginal tablet	Compressed or effervescent; mucoadhesive options (HPMC-based)	High (with slow-release excipients)	Accurate dosing; probiotic viability preserved	Insertion burden; effervescent tablets require moisture activation	Mesdaghinia 2025 [17]; Ravel 2025 [18]
Douche/Wash	Aqueous herbal solution; low viscosity	Low (transient)	Simple external or internal application	Very short contact time; limited deep vaginal penetration	Leite 2011 [19]; Moshfeghy 2018 [20]
Fumigation	Drug vaporization and diffusion; unique modality in traditional East Asian medicine	Low (transient mucosal exposure)	No insertion; culturally familiar in East Asia	Limited evidence base; external/vestibular effect only	Baek 2016 [21]; Park 2018 [22]

Abbreviations: HPMC, hydroxypropyl methylcellulose; PEG, polyethylene glycol; GSM, genitourinary syndrome of menopause; O/W, oil-in-water.

**Table 2:** Key clinical trials of herbal and natural intravaginal preparations.

Reference	Ingredient	Condition	Formulation	Design and n	Key Outcome
Simbar 2008 [11]	*Zataria multiflora*	BV	Cream	RCT, *n* = 90	Comparable clinical cure rates to metronidazole
Khosravi 2008 [12]	*Z. multiflora*	Acute VVC	Cream	RCT	White cheesy discharge positivity reduced from 100% to 13.8%
Zare 2019 [23]	*Quercus brantii*	BV	Cream	RCT	Significant reduction in Amsel criteria vs. placebo (*p* < 0.05)
Simbar 2026 [15]	*Quercus infectoria*	VVC	Suppository	Triple-blind RCT, *n* = 90	Comparable symptom relief to clotrimazole (*p* > 0.05); mean complete improvement time 2.5 ± 1.7 vs. 3.0 ± 1.5 days
Zeinab 2018 [20]	*Q. brantii*	VVC	Douche	RCT	Comparable antifungal outcomes to clotrimazole
Sritonchai 2020 [13]	*Pueraria mirifica*	GSM	Gel	RCT, *n* = 60	Abnormal Nugent scores 6.7% vs. 23.3% (*p* = 0.006); VHI increase 2.12 vs. 0.81 (*p* = 0.016)
Mohamadi 2025 [24]	Curcumin	BV	Cream	RCT	Greater complete improvement rate than metronidazole (82% vs. 42%, *p* < 0.001)
Baery 2018 [25]	Forzejeh (Persian herbs)	BV	Suppository	Double-blind RCT	Clue cell elimination 87.50% vs. 68.25% with metronidazole (*p* = 0.001)
Liu 2023 [26]	Fufang Furong	Mixed vaginitis	Effervescent suppository	Multicenter RCT, *n* = 559	Cure rate 63.51% vs. 67.15% (rate diff −3.64%); met non-inferiority margin (−12%)
Mesdaghinia 2025 [17]	Oregano + Lemon balm	BV	Vaginal tablet	Triple-blind RCT	Comparable improvement in Amsel criteria
Avijgan 2012 [27]	Echinophora platyloba	Recurrent VVC	Cream (adjunct)	RCT	Numerically lower treatment failure and long-term recurrence rates than fluconazole alone (20% vs. 43.3%; 26.7% vs. 56.7%)
Khazaeian 2018 [28]	Sucrose (Prebiotic)	BV	Gel	RCT	Symptom improvement comparable to metronidazole (85.7% vs. 88.5%, *p* = 0.389)
Galli 2024 [29]	*Zanthoxylum*, *Centella*, Tamarind	Vaginal atrophy	Medical device gel	Observational, 150 days	Vaginal dryness reduced by 18%, dyspareunia reduced by 14%

Abbreviations: BV, bacterial vaginosis; GSM, genitourinary syndrome of menopause; RCT, randomized controlled trial; VHI, Vaginal Health Index; VVC, vulvovaginal candidiasis.

**Table 3:** Key clinical trials of probiotic and combination vaginal preparations.

Reference	Strain(s)/Component	Formulation	Condition	Follow-Up	Key Outcome
Cohen 2020 [77]	*Lactobacillus crispatus* CTV-05 (Lactin-V)	Live biotherapeutic	BV	24 weeks	Cumulative recurrence lower than placebo (RR 0.66, 95% CI 0.44–0.87)
Larsson 2008 [78]	*L. gasseri* EB01, *L. rhamnosus* PB01	Vaginal capsule	BV	6 months	64.9% vs. 46.2% remained BV-free among initially cured women (*p* = 0.027)
Tomusiak 2015 [79]	*L. fermentum*, *L. plantarum*, *L. gasseri*	Vaginal capsule	Intermediate flora	14 days	Nugent score 2.12→0.9; 82% successful colonization
Parma 2013 [80]	*L. rhamnosus*	Vaginal tablet	Recurrent BV (surgical menopause)	12 months	57.1% (12/21) of high-risk patients remained completely recurrence-free
Ya 2010 [81]	Probiotic capsules	Vaginal capsule	Recurrent BV	2 months	Recurrence rate 15.8% vs. 45.0% (placebo) (OR 0.23, *p* < 0.001)
Rezazadeh 2024 [82]	Probiotic capsules	Vaginal vs. Oral	BV	—	Nugent score 8.5→3.0 (vaginal) vs. 9.0→3.0 (oral); no significant difference (*p* = 0.053)
Pane 2024 [83]	*L. fermentum* LF5	Vaginal product	VVC	2 weeks	Eradication 96% vs. 94% (miconazole); local adverse reactions 4% vs. 12%
Bradshaw 2012 [84]	Estrogen-containing vaginal probiotic	Vaginal gel	BV	6 months	No significant recurrence reduction vs. placebo (32% vs. 33%, *p* > 0.05)
Donders 2010 [85]	*L. acidophilus* + estriol 0.03 mg (Gynoflor^®^, Medinova AG, Zürich, Switzerland)	Vaginal tablet	BV	4–6 weeks	Short-term equivalence to metronidazole; inferior long-term suppression
Donders 2014 [86]	*L. acidophilus* + estriol 0.03 mg (Gynoflor^®^)	Vaginal tablet	Atrophy (breast cancer)	4 weeks	VMI 31%→70%; E2/E1 no systemic increase; local safety supported in aromatase inhibitor users
Murina 2018 [87]	Thymol, eugenol + *L. fermentum* + *L. plantarum* + GOS (Synbiotic)	Medical device	BV and VVC	—	Microbiological normalization reported in BV, VVC, and recurrent VVC subgroups (80.0%, 62.5%, and 100.0%, respectively)
Ravel 2025 [18]	*L. crispatus* LUCA103, LUCA011, LUCA009 + maltose + calcium lactate (Synbiotic VS-01)	Vaginal tablet (slow-release)	Vaginal dysbiosis	21 days	CST I conversion higher than placebo (90% vs. 11%, *p* < 0.002); slow-release vaginal tablet showed more favorable microbiome conversion than other delivery formats

Abbreviations: BV, bacterial vaginosis; CI, confidence interval; CST, community state type; E1, estrone; E2, estradiol; GOS, galactooligosaccharides; OR, odds ratio; RR, relative risk; VMI, vaginal maturation index; VVC, vulvovaginal candidiasis.

**Table 4:** Key phytochemicals and putative mechanisms for vaginal health.

Key Bioactive Components	Botanical Source	Putative Mechanism of Action	Target Condition
Carvacrol, Thymol, p-Cymene	*Zataria multiflora*	Broad-spectrum antibacterial and antifungal activity via cell membrane disruption; potential *Lactobacillus*-sparing effects	BV, VVC
Matrine, Oxymatrine	*Sophora flavescens*	Antimicrobial and anti-inflammatory effects; potential effects on vaginal microecological balance	BV, VVC, Trichomoniasis
Miroestrol, Deoxymiroestrol	*Pueraria mirifica*	Phytoestrogenic activity via estrogen receptor binding; improvement in vaginal microenvironmental parameters	GSM, Atrophic vaginitis
Trans-anethole	*Foeniculum vulgare*	Weak phytoestrogenic activity mitigating vaginal atrophy with limited reported systemic hormonal effects	Atrophic vaginitis
Tannins, Gallic acid, Ellagic acid	*Quercus* spp.	Antimicrobial and astringent properties; broad-spectrum activity against vaginal pathogens across multiple formulation types	BV, VVC
Thymol, Eugenol	*Thymus vulgaris, Eugenia* spp.	Local antimicrobial activity; potential *Lactobacillus*-sparing effects relevant to compatibility with probiotic vaginal formulations	BV, VVC, RVVC

Abbreviations: BV, bacterial vaginosis; GSM, genitourinary syndrome of menopause; RVVC, recurrent vulvovaginal candidiasis; VVC, vulvovaginal candidiasis.

**Table 5:** Evidence from systematic reviews and meta-analyses.

Reference	Intervention	Studies and Patients	Key Finding
Lan 2015 [14]	Kushen gel (*Sophora flavescens*)	4 RCTs	Greater symptom improvement vs. metronidazole (RR 1.13, 95% CI 1.06–1.20) with fewer adverse events (RR 0.29)
Wu 2025 [126]	Topical Chinese polyherbal preparations (NMA)	9 preparations	*S. flavescens*-based preparation ranked 4th overall in negative conversion rates among 9 CCPPs
Park & Jo 2019 [127]	Traditional East Asian herbal medicine	26 RCTs, 3162 patients	Reported favorable effects for atrophic vaginitis; potential to decrease recurrence rates vs. conventional therapies
Najafi 2019 [5]	Herbal medicines for BV	13 RCTs	*Zataria multiflora* showed outcomes comparable to metronidazole; garlic preparations showed favorable effects in selected endpoints
Kamali 2024 [130]	Medicinal plants for BV	20 RCTs, 2685 patients	Herbal-antibiotic combinations may show favorable outcomes; *Zataria* and *Calendula* reported comparable outcomes to conventional therapy
Baek 2016 [21]	Fumigation therapy for atrophic vaginitis	5 RCTs	Clinical improvement trends reported; heat-mediated local exposure proposed as a possible mechanism
Ma 2023 [131]	Antibiotics + intravaginal probiotics	11 RCTs, 1493 patients	Significant short-term BV recurrence reduction at 12–16 weeks (RR 0.11); long-term benefits require further study
Senok 2009 [1]/Xie 2017 [8]	Probiotics for BV/VVC (Cochrane)	Cochrane reviews	Insufficient evidence for probiotics as standalone primary therapy; supports further evaluation of formulation-optimized probiotic or synbiotic approaches

Abbreviations: BV, bacterial vaginosis; CCPPs, Chinese compound prescription preparations; CI, confidence interval; NMA, network meta-analysis; RCT, randomized controlled trial; RR, risk ratio; VVC, vulvovaginal candidiasis.

## Data Availability

No new data were created or analyzed in this study. Data sharing is not applicable.

## Abbreviations

The following abbreviations are used in this manuscript:

BV	bacterial vaginosis
CI	confidence interval
CNKI	China National Knowledge Infrastructure
GSM	genitourinary syndrome of menopause
KM	Korean medicine
RCT	randomized controlled trial
RR	relative risk
TCM	Traditional Chinese medicine
VVC	vulvovaginal candidiasis
O/W	oil-in-water
HPMC	hydroxypropyl methylcellulose
PEG	polyethylene glycol
OASIS	Oriental Medicine Advanced Searching Integrated System
VHI	vaginal health index
CST	community state type
GOS	Galactooligosaccharides
NMA	network meta-analysis
CCPPs	Chinese compound prescription preparations
RVVC	recurrent vulvovaginal candidiasis
VMI	vaginal maturation index
E1	estrone
E2	estradiol
